# CDH4 inhibits ferroptosis in oral squamous cell carcinoma cells

**DOI:** 10.1186/s12903-023-03046-3

**Published:** 2023-05-27

**Authors:** Jian Xie, Ting Lan, Da-Li Zheng, Lin-Can Ding, You-Guang Lu

**Affiliations:** 1 Department of Preventive Dentistry, School and Hospital of Stomatology, Fujian Medical University, 246 Yang Qiao Middle Road, Fuzhou, 350002 China; 2 Key Laboratory of Stomatology of Fujian Province, School and Hospital of Stomatology, Fujian Medical University, 88 Jiaotong Rd, Fuzhou, 350004 China

**Keywords:** CDH4, R-cadherin, Ferroptosis, Oral squamous cell carcinoma

## Abstract

**Background:**

The cadherin-4 gene (CDH4), a member of the cadherin family genes, encodes R-cadherin (R-cad); however, the function of this gene in different types of cancer remains controversial. The function of CDH4 in OSCC (oral squamous cell carcinoma) is unknown.

**Materials and methods:**

We use the Cancer Genome Atlas (TCGA) database to find the expression of CDH4 in OSCC is more than normal tissue. Our tissue samples also confirmed that CDH4 gene was highly expressed in OSCC. The related cell function assay detected that CDH4 promotes the ability of cell proliferation, migration, self-renewal and invasion. Cell staining experiment confirmed that the change of CDH4 expression would change the cell mortality. The western blot of GPX4 (glutathione-dependent peroxidase-4), GSH (reduced glutathione) test assay and MDA(Malondialdehyde) test assay show that the expression of CDH4 may resist the sensitivity of ferropotosis in OSCC.

**Results:**

CDH4 was upregulated in OSCC samples and was correlation with poor survival of patients. High expression of CDH4 effectively promotes the proliferation, mobility of OSCC cells and reduce the sensitivity of OSCC cells to ferroptosis. CDH4 is positively correlated with EMT pathway genes, negatively correlated with fatty acid metabolism pathway genes and peroxisome pathway genes, and positively correlated with ferroptosis suppressor genes in OSCC.

**Conclusions:**

These results indicate that CDH4 may play a positive role in tumor progression and resistance ferroptosis and may be a potential therapeutic target for OSCC.

**Supplementary Information:**

The online version contains supplementary material available at 10.1186/s12903-023-03046-3.

## Introduction

The cadherin family consists of classical intercellular adhesion molecules that are Ca2 + -dependent [1, 2]. The extracellular domain of cadherin binds to catenin, and is connected to the cytoskeleton, thus forming cell–cell adhesions (AJs). AJs is the most common and universal type of cell adhesion [3]. Therefore, cadherin is necessary for the stability of intercellular adhesion and normal cell physiological activities. It is related to cell–cell adhesion, cell growth, cell polarity, cell motility, differentiation and survival [4–6]. When cells undergo malignant transformation, the destruction of cell adhesion will help tumor cells to invade and metastasize. Some studies believe above changes must be related to cadherin, AJs or activating some signal pathways [7–12]. CDH4 is a member of the cadherin family and encodes R-cadherin. CDH4 plays an important role in the differentiation of retina, kidney, striated muscle and brain nerves [13–17]. It also is found to be associated with the occurrence and metastasis of a variety of tumors, but its function in different types of cancer is controversial [18–27].

Ferroptosis is a new cell death pattern discovered by Dixon in 2012 [28]. It is a programmed cell death, which is mainly caused by lipid peroxidation of mitochondrial membrane due to iron-dependent ROS (reactive oxygen species) accumulation in cells [29]. Ferroptosis is considered to have three classical pathways, GPX4 regulation pathway, iron metabolism pathway and lipid metabolism pathway [30]. Ferroptosis has been found in many kinds of cancer and is considered as the key mechanism of tumor treatment research [31, 32]. Tumor cells require more iron to maintain vigorous growth than normal cells, ferroptosis is considered to be an adaptive mechanism to eliminate malignant cells, especially in tumor cell lines that have developed resistance to chemotherapy drugs [33–35].

OSCC is a common cancer worldwide, especially in South Asia. It is an important part of HNSCC (head and neck squamous cell carcinoma). There were approximately 377,713 new cases of OSCC and 17,757 new deaths in 2021. OSCC is the eighth most common cancer among men, and it has the highest incidence in Asia [36]. New cases of OSCC account for 3% of all new cancer cases, and deaths from OSCC account for 1.8% of all deaths in the United States [37]. OSCC has a poor five-year overall survival rate of 40%-50%, and the survival rate of advanced cancer patients is only 20% [38, 39]. The conventional treatment of OSCC is surgery, radiation therapy and chemotherapy, often combination therapy. The variability of patients' response to OSCC treatment is relatively high, and the recurrence rate of OSCC is as high as 50% [40]. Current treatments are frequently ineffective and severely affect patient quality of life [41].

Despite the development of study and treatments, overall survival has not improved significantly over the past 20 years [42]. The poor prognosis of this disease is due to a propensity for recurrence and distant metastasis [43]. At present, for the treatment of OSCC, some studies start with precancerous lesions [44, 45], some study the application of compound Chinese medicine [46], some study the optimization of chemotherapy drug delivery routes [47], and some are keen to find molecular targets for the diagnosis and treatment of oral cancer [48–50].however, the function of CDH4 gene in OSCC and its relationship with ferroptosis are unknown. In this study, we investigated the role of CDH4 and its relationship with ferroptosis in OSCC. To study the possibility of CDH4 gene as a target for diagnosis and treatment.

## Materials and methods

### Cell lines and cell culture

The CAL27, HN30, HN6 and SCC9 cell lines were purchased from ATCC (American Type Culture Collection, USA). CAL27, HN30 and HN6 cells were grown in high-glucose DMEM (Gibco BRL, Grand Island, NY, USA) supplemented with 10% FBS (Gibco BRL, Grand Island, NY, USA). SCC9 cells were grown in DMEM/F-12 (Gibco BRL, Grand Island, NY, USA) supplemented with 10% FBS. All cells were incubated in a humidified atmosphere containing 5% CO2 at 37 °C.

### Acquisition of tissue samples

Fresh samples of 22 patients with oral squamous cell carcinoma and normal tissues adjacent to cancer were obtained from the First Affiliated Hospital of Fujian Medical University. According to the guidelines of Helsinki Declaration, the behavior was informed by each patient and provided with a written informed consent. The study was approved by the Ethics Committee of the Fujian Medical University, ethical number 2021-FMUSS-024.

### Real-time quantitative RT–PCR (qPCR)

Nucleozol Reagent (Invitrogen, USA) was used to extract total RNA from 3 × 105 HN30 and SCC9 cells cultivated in six-well plates. Total RNA was reverse transcribed to cDNA using the PrimeScript™ RT reagent kit (Takara, Japan). Real-time PCR was performed with SYBR GREEN Supermix (BIO-RAD, USA) using the following program: initial denaturation at 94 °C for 10 min, followed by 40 cycles of 94 °C for 15 s and 60 °C for 45 s. Data were analyzed according to the 2- ΔΔCT method. The sequences of CDH4 and ACTB are listed in Table 1.

**Table 1:** The primers for real-time PCR

Gene	Accession no	Forward	Reverse
ACTB	NM001101	CCTGGCACCCAGCACAAT	GGGCCGGACTCGTCATACT
CDH4	NM001794.2	CGTCCATCATCAAAGTCAAGGT	GGTCGTAGTCCTGGTCCTCCT

### Design and synthesis of corresponding siRNAs and CDH4 overexpression vector

To target different coding regions of CDH4, three corresponding siRNAs were designed and synthesized (Shanghai GenePharma Co., Shanghai, China). The sequence information is presented in Table 2. All siRNAs were separately transfected into HN30 and SCC9 cells using Lipofectamine® RNAmate Transfection Reagent (Invitrogen, USA) following the manufacturer’s instructions. The pcDNA3.1-FLAG-CDH4 plasmid and pcDNA3.1-vector plasmid was designed and synthesized by (Shanghai GenePharma Co., Shanghai, China). were separately transfected into HN30 and SCC9 cells using Lipofectamine™ 2000 Transfection Reagent (Invitrogen, USA).

**Table 2:** The siRNA and negative control sequences for CDH4

Name	Sequence	
siRNA-1	5′‐CGUCCAUCAUCAAAGUCAATT‐3′	5′‐UUGACUUUGAUGAUGGACGTT‐3′
siRNA-2	5’-GCGACAACAUCCUCAAGUATT-3’	5’-UACUUGAGGAUGUUGUCGCTT-3’
NC	5’-UUCUCCGAACGUGUCACGUTT-3’	5’-ACGUGACACGUUCGGAGAATT-3’

### Western blotting

Total protein was separated by 8% SDS–PAGE and transferred onto PVDF membranes (Amersham, USA). The membranes were blocked in 1% bovine serum albumin and incubated with primary antibodies against R-cad (Abnova Cat# PAB1864) at a dilution of 1:500 at 4 °C for 10 h, Gapdh (Cell Signaling Technology Cat# 51,332) at a dilution of 1:1000 at 4 °C for 10 h, β-actin (Cell Signaling Technology Cat# 5125) at a dilution of 1:1000 at 4 °C for 10 h, Flag and GPX4 (Cell Signaling, USA, Cat#52,455) at a dilution of 1:1000 at 4 °C for 10 h. When the expected bands, estimated by the molecular weight ladder and the manufacturer’s instructions of primary antibodies, were separated enough, the blots were cut into 2 or 3 parts prior to incubation with primary antibodies. The original gels and multiple exposure images were shown in Supplementary Fig. 1.

### TCGA database, Hallmark gene set and ferroptosis database

The transcript data and clinical information from the HNSCC and OSCC datasets were downloaded from the TCGA database (http://gdc.cancer.gov). The data were obtained on March 8th, 2023, and the HNSCC dataset contained 504 HNSCC samples and 44 normal tissues adjacent to cancer. The OSCC samples contained 330 cancer samples and 32 normal tissues adjacent to cancer. Hallmark gene set from MSigDB (Molecular signatures database) was used to GESA to find the related genes and pathways. The data of ferroptosis gene comes from the ferroptosis database (zhounan.org/ferrdb).

### In vitro cell proliferation assay

Cell proliferation was evaluated by colony formation assay and counting of viable cells using the Cell Counting Kit-8 (CCK-8, Dojindo, Kumamoto, Japan). In the colony formation assay, 3 × 103 cells (CDH4 knockdown experinment) and 1.5 × 103 cells (CDH4 overexpression experiment) were plated in each well of a 6-well plate and cultivated for 2 weeks. After forming visible clones, the cells were stained with crystal violet, and the number of clones was counted. The experiments were conducted in triplicate and repeated three times. In the CCK-8 assay, first, the cells were transfected with siRNA and then plated into a 96-well plate at a rate of 3 × 103 per well and CDH4 overexpression cells plated into a 96-well plate at 1.5 × 103 per well. The wells were monitored daily and observed continually for 6 or 7 days. CCK-8 reagent (10 µl) was added to the medium, and the plates were incubated for 1 h. The absorption at 450 nm was measured at the same time every day for 6 consecutive days.

### In vitro cell invasion assay

Cell invasion was evaluated by a transwell invasion assay using 24-well Matrigel-coated Transwell chambers (8-μm pore size, BD, USA). The HN30 cells were evenly plated at 1 × 10^5^ cells/well in DMEM without FBS in the upper chamber and DMEM containing 10% FBS in the lower chamber in CDH4 knockdown experiment. The HN30 cells were evenly plated at 8 × 10^5^ cells/well in DMEM without FBS in the upper chamber in CDH4 overexpression experiment. The SCC9 cells were plated at 5 × 104 cells/well in the upper chamber. After incubation at 37 °C for 48 h, Matrigel and cells on the upper chamber were removed with cotton swabs, and the cells were stained with crystal violet for 10 min. Cells were counted and photographed under a microscope in at least five random fields.

### In vitro cell migration assay

Cell migration was evaluated using transwell migration and wound-healing assays. The method of the transwell migration assay was similar to that of the transwell invasion assay, but the transwell chambers without Matrigel (8-μm pore size, BD, USA). In the upper chamber, HN30 cells were plated at 8 × 10^4^ cells/well in CDH4 knockdown experiment and plated at 6 × 10^4^ cells/well in CDH4 overexpression experiment, and SCC9 cells were plated at 4 × 10^4^ cells/well. For wound-healing assays, the transfected cells were plated in a 12-well plate, and when the cells were spread onto the bottom of the dish, a trace was drawn evenly. After scratching, the culture medium was changed to serum-free DMEM. Gap areas were photographed at different time points using a light microscope (40X).

### Cancer stem cell sphere culture

OSCC cells were collected, and serum was removed. HN30 cells were suspended in serum-free DMEM and stem cell medium (STEMCELL Technologies Inc., Catalog # 05926) at a ratio of 1:1. The cells were cultured in 96-well plates in ultralow attachment dishes (Corning Life Sciences, Catalog#3262). HN30 cells were seeded at a density of 1000 cells/100 µl in CDH4 knockdown experiment and seeded at a density of 500 cells/100ul in CDH4 overexpression experiment. After two weeks of cultivation, the spheres of cells were statistically analyzed.

### Live and dead cell staining

Live and dead cell staining was performed using a Calcein-AM/PI assay kit (Solarbio, China). Treated cells were stained with calcein-AM solution (1:1000) and incubated at 37℃ for 20 min, then added PI solution (3:1000) incubated at 37℃ for 5 min. The numbers of live and dead cells were detected under a laser confocal scanning microscope (OLYMPUS IX83, USA). The live cells fluoresced green (Calcein-AM staining), and the dead cells fluoresced red (PI staining). Under 400X magnification, 6 fields of view were taken for each hole.

### GSH/GSSG detection experiment and MDA test assay

GSH/GSSG in cells was detected by a GSH and GSSG Assay Kit (Beyotime, China). Cells transfected for 48 h were treated with this kit and added to 96-well plate incubated for 30 min to detect GSH. The absorption was detected at 412 nm. MDA is tested by MDA test assay kit (Beyotime, China). Cells transfected for 48 h were treated with extracting solution in ultrasonic 7 min (work for 3 s and rest for 10 s in each time). The supernatant of cells was obtained after centrifugation 10,000 rpm 10 min. The supernatant weas added corresponding reagents, incubated at 100℃ for 1 h and centrifugated at 8000 rpm 10 min. The final supernatant was added 200ul to each well into 96-well plate.

The absorption was detected at 532 nm and 600 nm.

### Statistical analysis

The experimental data were analyzed and graphed by GraphPad Prism 9 (GraphPad Software, USA). Paired Clinical samples were tested by Wilcoxon signed rank test and unpaired samples were tested by Wilcoxon rank sum test. Overall survival was tested by Km plot log-rank test. Genes correlation analysis was tested by person or spearman test. Comparisons between two groups were performed using two tailed unpaired t test and comparisons among three groups were performed using one-way analysis of variance. In the graphs, ns indicated *P* > 0.05, while *P* < 0.05 was considered statistically significant (* indicates *P* < 0.05, **indicates *P* < 0.01, ***indicates *P* < 0.001, ****indicates *P* < 0.0001, Values are presented as means ± standard error.).

## Results

### CDH4 is upregulated in OSCC

In order to study the function of CDH4 in OSCC, we used the data of tumor samples given by TCGA to analyze the expression of CDH4 in different tissues and the relationship between CDH4 expression and overall survival rate of patients. OSCC is an important part of head and neck squamous cell carcinoma (HNSCC), we have analyzed the samples from HNSCC and OSCC, hoping to get hints through as many samples as possible. After the database sample analysis, we also analyzed the samples of 22 patients' OSCC tissues and normal tissues adjacent to cancer obtained from the First Affiliated Hospital of Fujian Medical University to further clarify the expression of CDH4 in OSCC.

**Fig. 1:**
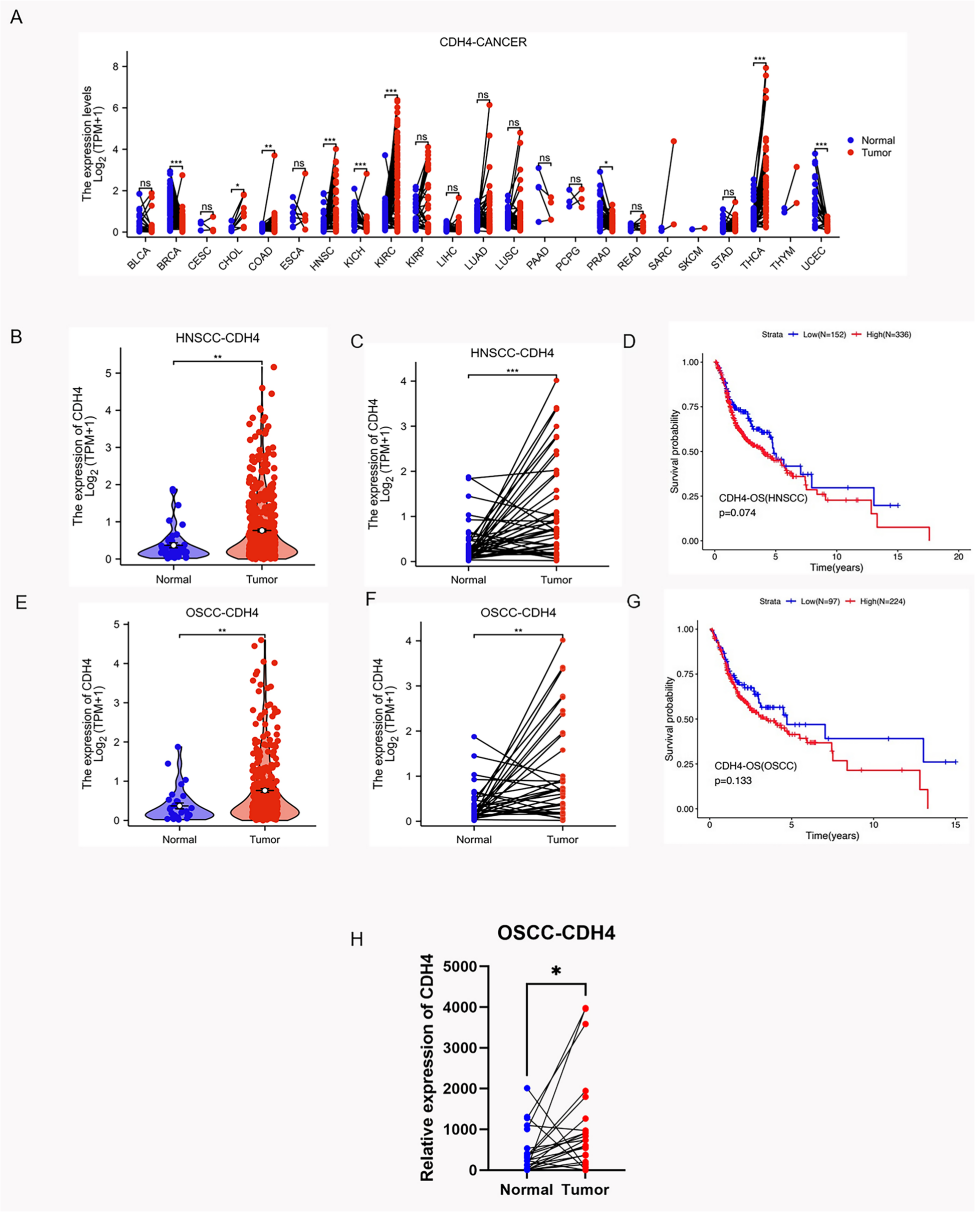
CDH4 is upregulated in OSCC. The expression of CDH4 is different in many kinds of cancers in the TCGA database (A). Compared with normal tissues adjacent to cancer, CDH4 is highly expressed in HNSCC tissues in unpaired samples (B, *P* < 0.01) and paired samples (C, *P* < 0.001). The overall survival rate was higher in the CDH4-low-expression group than in the CDH4-high-expression group (D, *P* = 0.074). CDH4 is highly expressed in OSCC tissues in unpaired samples (E, *P* < 0.01) and paired samples (F, *P* < 0.01). The overall survival rate was higher in the CDH4-low-expression group than in the CDH4-high-expression group (G, *P* = 0.133). CDH4 is highly expressed in our clinical OSCC paired samples (H, *P* < 0.05). (* indicates *P* < 0.05, **indicates *P* < 0.01, ***indicates *P* < 0.001, ****indicates *P* < 0.0001)

The chart shows the differential expression of CDH4 between cancer and normal tissues adjacent to the cancer in the TCGA database (Fig. 1A). We found that the expression of CDH4 is higher in cholangiocarcinoma (CHOL), Colon adenocarcinoma (CAOD), KIRP (Kidney renal papillary cell carcinoma), KIRC (Kidney renal clear cell carcinoma), THCA (Thyroid carcinoma) and HNSCC than normal tissues adjacent to the cancer. In HNSCC, the CDH4 is more highly expressed than in adjacent normal tissues (Fig. 1B). More highly expressed in cancer tissues than in normal adjacent cancer tissues in 43 paired samples (Fig. 1C). The median overall survival of patients, in the CDH4-high-expression group was shorter than that in the CDH4-low-expression group in HNSCC, but the difference is not significant enough (Fig. 1D). In OSCC, the CDH4 is more highly expressed in cancer tissues than in normal tissues (Fig. 1E) and more highly expressed in cancer tissues than in normal adjacent cancer tissues in the same patient (Fig. 1F) in 32 paired samples. The median overall survival of patients, in the CDH4-high-expression group was shorter than that in the CDH4-low-expression group in OSCC, but the difference is not significant enough (Fig. 1G). We extracted RNA from fresh clinical OSCC samples and detected the expression of CDH4 in the samples by real-time PCR. The expression of CDH4 is higher in OSCC group than in normal adjacent cancer tissues in 22 paired samples of our OSCC samples (Fig. 1H). We analyzed the expression of CDH4 in HNSCC and OSCC in the database, and combined with the analysis of CDH4 in our own tissue samples, we found that the expression of CDH4 is higher in cancer tissues than in normal tissues adjacent to cancer, which may remind that CDH4 promotes the occurrence and development of OSCC.

### Downregulation of CDH4 expression suppressed the proliferation and stemness of OSCC cells in vitro

**Fig. 2:**
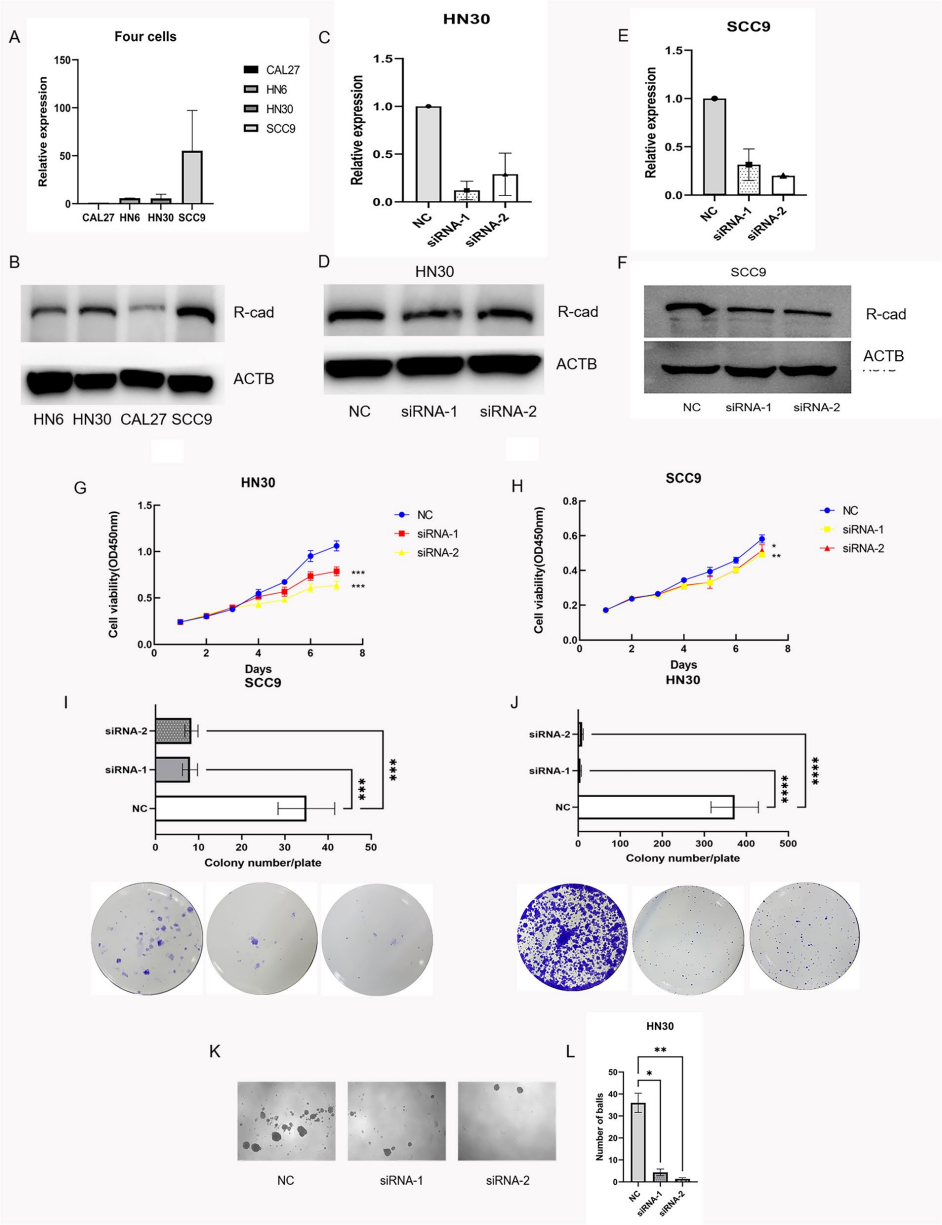
Knockdown of CDH4 suppresses the proliferation and stemness of OSCC. The expression of CDH4 in four kinds of OSCC cells **A**-**B**. CDH4 expression was effectively reduced by siRNAs as measured by real-time PCR **C** and western blot **D** in HN30 cells. CDH4 expression was effectively reduced by siRNAs as measured by real-time PCR **E** and western blot **F** in SCC9 cells. After transfection with CDH4 siRNAs, the growth of HN30 cells **G** and SCC9 **H** cells was measured by CCK-8 assay. The colony formation assay showed that the proliferation of HN30 cells **I** and SCC9 cells **J** was decreased after CDH4 knockdown. The number of colonies was counted. The picture shows the cell sphere formation in different groups under a 40X microscope **K**, and the number of spheres was decreased in the siRNA-1 and siRNA-2 groups compared with the NC group **L**. These experiments were repeated three times. (* indicates *P* < 0.05, **indicates *P* < 0.01, ***indicates *P* < 0.001, ****indicates *P* < 0.0001, Values are presented as means ± standard error.)

Firstly, we investigated whether CDH4 is related to the proliferation and self-renewal ability of OSCC. Among the four OSCC cell lines, higher expression levels of CDH4 were seen in the HN30 and SCC9 cell lines than in the HN6 and CAL27 cell lines (Fig. 2A-B). To detect the function of CDH4 in the proliferation of OSCC cells, two siRNAs targeting CDH4 were transfected into SCC9 and HN30 cells to knockdown CDH4 expression. Realtime-PCR and western blotting showed that the CDH4 gene was significantly downregulated in HN30 (Fig. 2C-D) and SCC9 cells (Fig. 2E-F). Changes in cell proliferation ability were observed after downregulation of CDH4. CCK-8 assays and plate colony formation experiments confirmed that cell proliferation in the siRNA-1 and siRNA-2 groups was lower than that in the NC group in HN30 cells (Fig. 2G,I) and SCC9 cells (Fig. 2H,J). Cell spheroidization experiments showed that the self-renewal of cells was decreased in the siRNA-1 and siRNA-2 groups compared with the NC group in HN30 cells (Fig. 2K-L). Downregulation of the CDH4 gene inhibits the proliferation and self-renewal of OSCC cells in vitro.

### Downregulation of CDH4 expression suppresses OSCC migration and invasion in vitro

**Fig. 3:**
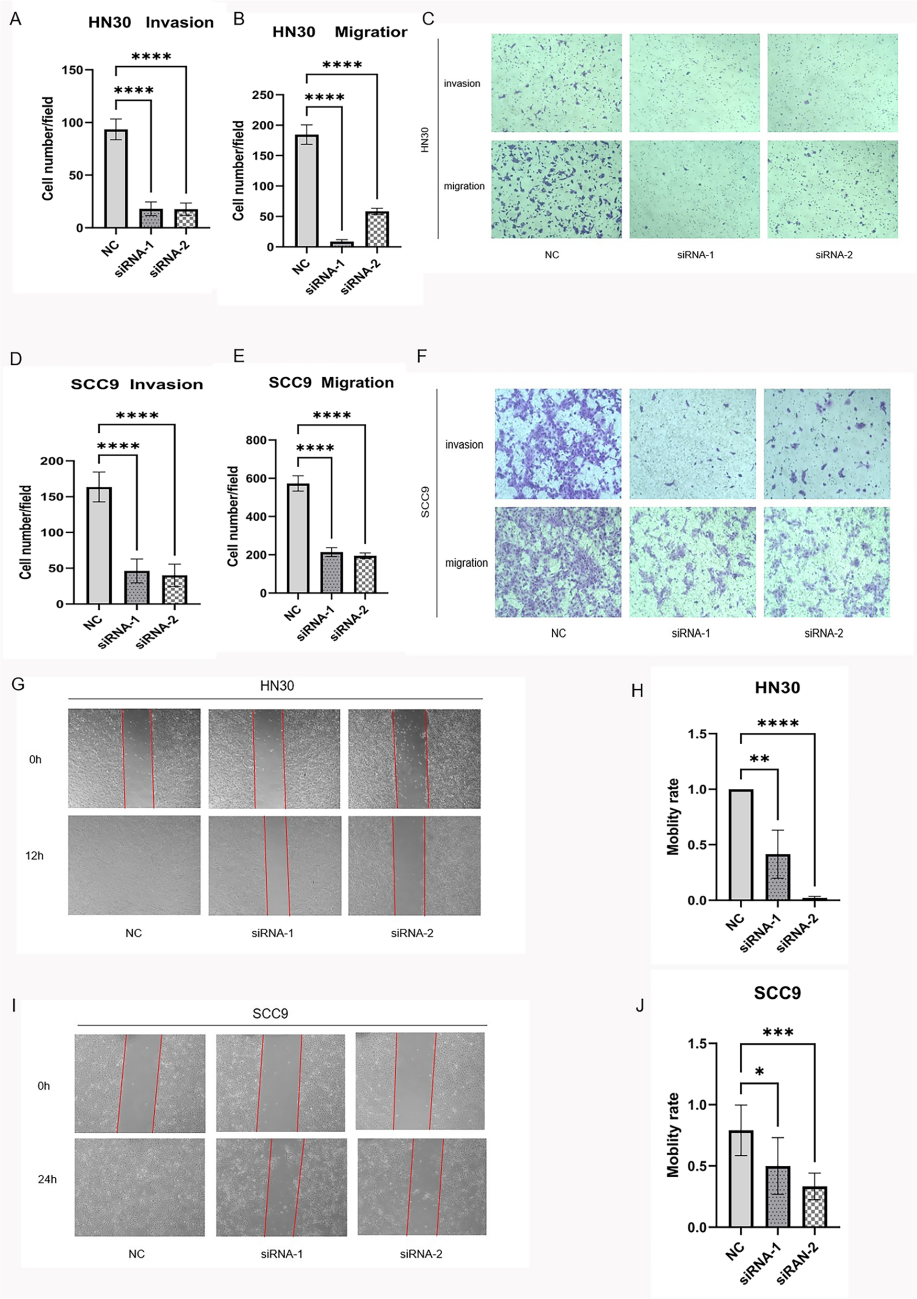
Knockdown of CDH4 suppresses the motility of OSCC. In the transwell migration assay and transwell invasion assay, the number of cells that passed through the filters was counted for HN30 cells **A**-**B** and SCC9 cells **D**-**E**. Representative images show the cells transfected with CDH4 that passed through the upper chamber of the transwell with and without (upper panel) Matrigel coating for HN30 cells **C** and SCC9 cells **F**. The numbers of migrating and invading cells were counted from at least five randomly selected fields of view under a microscope at 100X magnification. For the wound-heal assay, the representative images show the distances of cells passing through the scratch within a period of time for HN30 cells **G** and SCC9 cells **I** under a 40X microscope field of view. The mobility rate of cells was measured for HN30 cells **H** and SCC9 cells **J**. These experiments were repeated three times. (* indicates *P* < 0.05, **indicates *P* < 0.01, ***indicates *P* < 0.001, ****indicates *P *< 0.0001, Values are presented as means ± standard error.)

We found that cell mobility and invasion ability were weaken after siRNA downregulated the expression of CDH4. In the transwell migration assay and transwell invasion assay, the numbers of cells that passed through the monolayers of the upper panel of transwells without Matrigel and transwells with Matrigel in the siRNA-1 and siRNA-2 groups were lower than that in the NC group in HN30 cells (Fig. 3A-C) and SCC9 cells (Fig. 3D-F). Furthermore, wound healing assays showed that the mobility of cells in the siRNA-1 and siRNA-2 groups was slower than that in the NC group in HN30 cells (Fig. 3G-H) and SCC9 cells (Fig. 3I-J). Downregulation of the CDH4 gene inhibits the mobility of OSCC cells in vitro.

### Overexpression of CDH4 improved the proliferation, mobility and stemness of OSCC cells in vitro

**Fig. 4:**
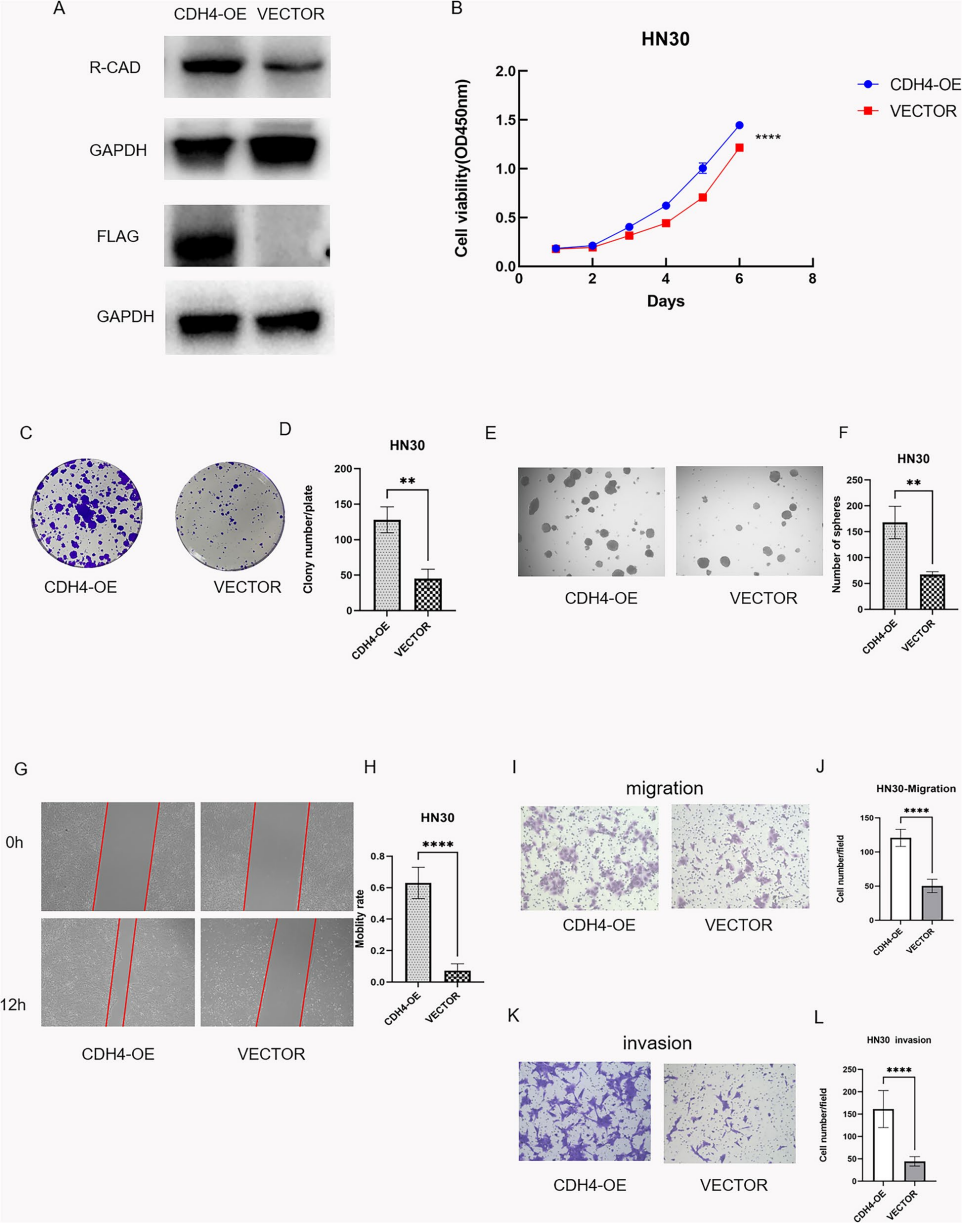
Overexpression of CDH4 improved the proliferation, stemness and motility of OSCC. CDH4 expression was effectively increased by pcDNA3.1-FLAG-CDH4 plasmid as measured by western blot **A**. The growth of HN30 cells was measured by CCK-8 assay **B**. The colony formation assay showed that the proliferation of HN30 cells was increased after CDH4 up-regulation **C**. The number of colonies was counted **D**. The picture shows the cell sphere formation in different groups under a 40X microscope **E**, and the number of spheres was increased in the CDH4-overexpression group compared with the vector group **F**. For the wound-heal assay, the representative images show the distances of cells passing through the scratch within a period of time for HN30 cells **G** under a 40X microscope field of view. The mobility rate of cells was measured for HN30 cells **H**. In the transwell migration assay, representative images show the cells transfected that passed through the upper chamber of the transwell without (upper panel) Matrigel coating for HN30 cells **I**. The number of cells that passed through the filters was counted for HN30 cells **J**. In the transwell invasion assay, representative images show the cells transfected that passed through the upper chamber of the transwell with (upper panel) Matrigel coating for HN30 cells **K**. The number of cells that passed through the filters was counted for HN30 cells **L**. The numbers of migrating cells were counted from at least five randomly selected fields of view under a microscope at 100X magnification. These experiments were repeated three times. (* indicates *P* < 0.05, **indicates *P* < 0.01, ***indicates *P* < 0.001, ****indicates *P* < 0.0001, Values are presented as means ± standard error.)

After the functional experiment of CDH4-down-regulation, we up-regulated the expression of CDH4 and studied the changes of cell functions. Changes in cell proliferation ability and mobility were observed after up-regulated the expression of CDH4. The expression of CDH4 was up-regulation in CDH4-overexpression group than vector group (Fig. 4A). CCK-8 assays and plate colony formation experiments confirmed that cell proliferation in the CDH4-overexpression group was higher compared with the vector group in HN30 cells (Fig. 4B-D). Cell spheroidization experiments showed that the self-renewal of cells was increased in the CDH4-overexpression groups compared with the vector group in HN30 cells (Fig. 4E-F). In wound healing assays showed that the mobility of cells in the CDH4-overexpression groups was faster than that in the vector group in HN30 cells (Fig. 4G-H). Furthermore, in the transwell migration assay and transwell invasion assay, the numbers of cells that passed through the monolayers of the upper panel of transwells without and with Matrigel in the CDH4-overexpression groups were more than that in the vector group in HN30 cells (Fig. 4I-L). Up-regulation of the CDH4 gene improves the proliferation, self-renewal and mobility of OSCC cells in vitro.

Through the above cytological function experiments, we found that CDH4 gene can promote cell proliferation, self-renewal, invasion and migration. We began to observe the reason why CDH4 gene can change the cell function, and whether the change of cell proliferation is related to the occurrence of ferroptosis.

### CDH4 can resist the sensitivity of OSCC cells to ferroptosis

**Fig. 5:**
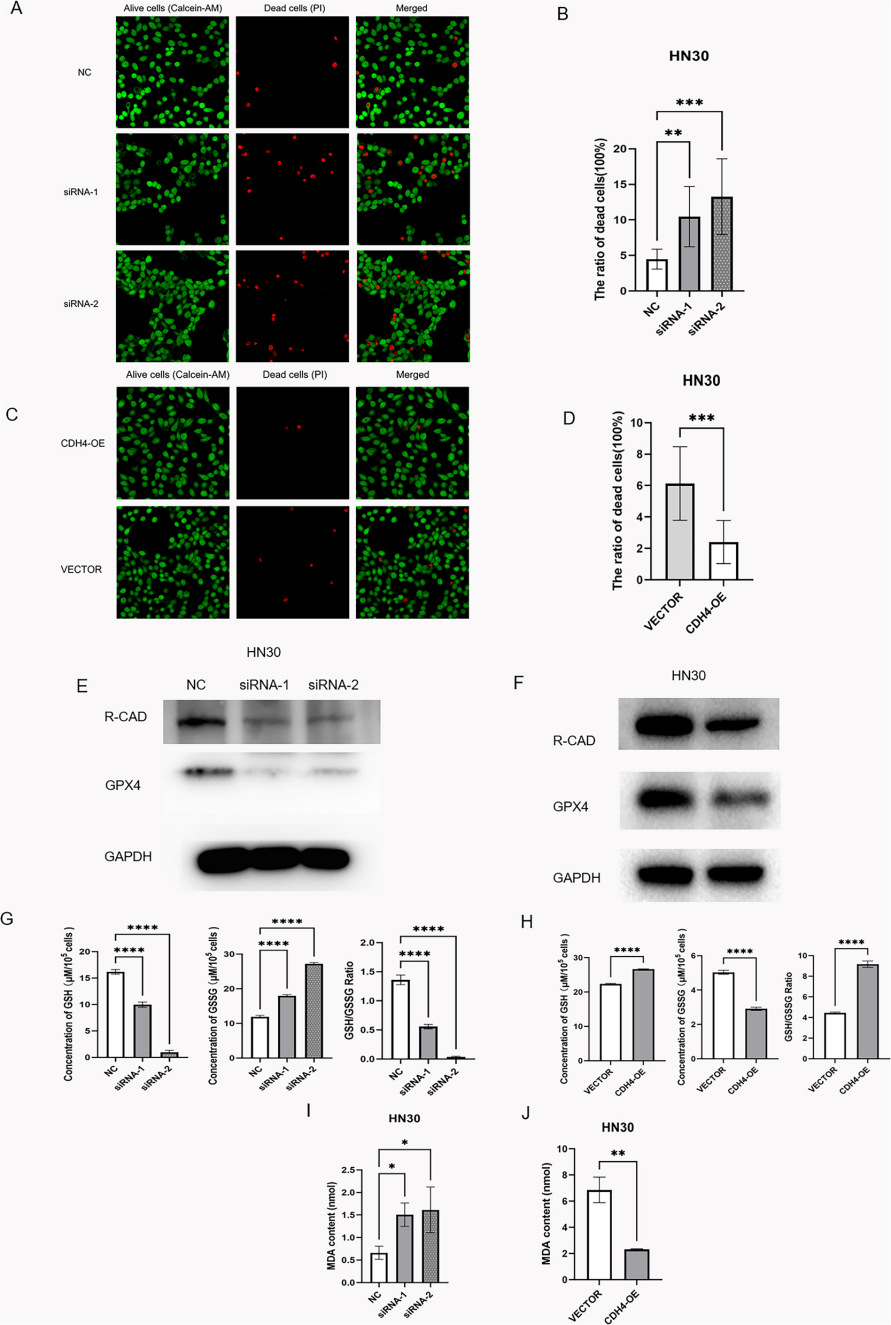
CDH4 can reduce the sensitivity of OSCC cells to ferroptosis. In the representative images, live cells fluoresced green (Calcein/AM) and dead cells fluoresced red (PI) **A**, **C**. The images include at least five randomly selected fields of view under a 400X laser confocal microscope. The statistical graph shows the percentage of dead cells **B**, **D**. The western blot experiment showed the expression of GPX4 in the CDH4 down-regulation experiment and CDH4 up-regulation experiment **E**–**F**. The expression of GSH, GSSG and the ratio of GSH/GSSG in the CDH4 down-regulation experiment and CDH4 up-regulation experiment **G**-**H**. The MDA in the CDH4 down-regulation experiment and CDH4 up-regulation experiment (Fig. I-J). (* indicates *P* < 0.05, **indicates *P* < 0.01, ***indicates *P* < 0.001, ****indicates *P* < 0.0001, and Values are presented as means ± standard error.)

The cell staining sassy showed that the mortality rate of HN30 cells is dramatically increased by downregulation of CDH4 and decreased by up-regulation of CDH4 (Fig. 5A-D). Through westernblot experiments, we found that compared with the NC control group, the GPX4 in siRNA-1 and siRNA-2 groups down-regulated by CDH4 decreased (Fig. 5E). Compared with vector group, the expression of GPX4 in CDH4-overexpression group was higher (Fig. 5F). Ferroptosis is due to the inactivation of antioxidant system in cells, which leads to the accumulation of lipid hydroperoxide in cells and eventually leads to cell death [51]. The system xc − glutathione (GSH)-GPX4-dependent antioxidant defense is the most important [52]. GPX4 is an antioxidant enzyme. When ferroptosis in GPX4 path, reduced GSH will become GSSG with the help of GPX4 [53]. After discovering the change of GPX4, we detected the change of GSH/GSSG. Compared with NC group, GSH in siRNA-1 and siRNA-2 groups decreased, GSSG increased and GSH/GSSG ratio decreased(Fig. 5G). Compared with vector group, GSH increased, GSSG decreased and GSH/GSSG ratio increased in CDH4-overexpression group (Fig. 5H). When oxygen free radicals react with unsaturated fatty acids of lipids to produce lipid peroxide as a by-product, we detect the level of lipid oxidation by detecting the level of MDA. The MDA experiment proved that the MDA produced by cells increased after the down-regulation of CDH4 expression, and decreased after the up-regulation of CDH4 expression (Fig. 5I-J). The above experiments confirmed that CDH4 can resist the sensitivity of cells to ferroptosis and suggest that the effect of CDH4 on cell proliferation may be due to its resistance to ferroptosis of cells.

### CDH4-related pathway analysis

**Fig. 6:**
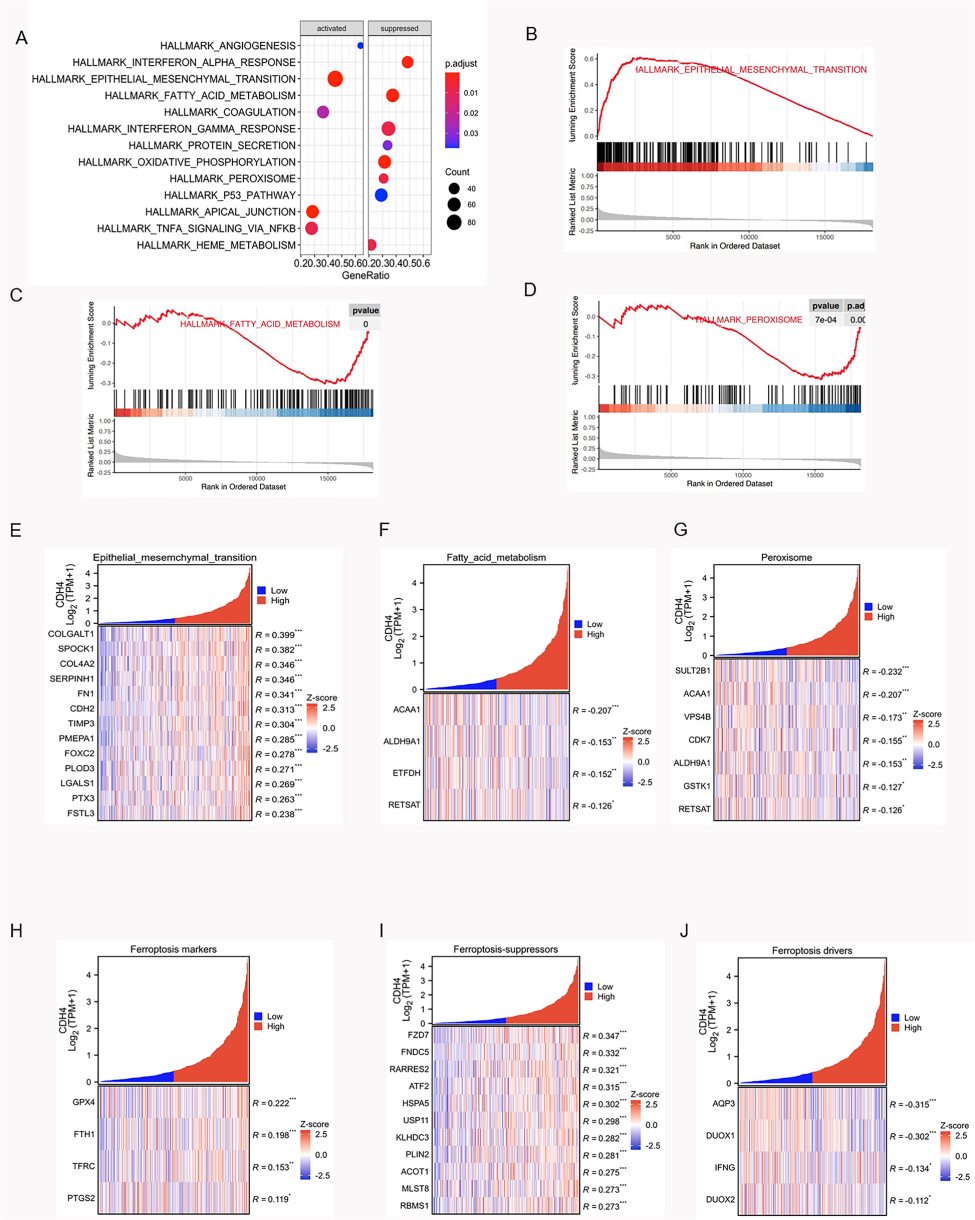
GSEA of CDH4 in the Hallmark gene set. Top ten related CDH4 pathways by GSEA hallmark enrichment analysis in HNSCC **A**. The EMT pathway is positively correlated with CDH4, fatty acid metabolism pathway and peroxisome pathway is negatively correlated with CDH4 in HNSCC **B**-**D**. Correlation analysis showed that EMT pathway gene was related to CDH4 in OSCC **E**. Correlation analysis showed that fatty acid metabolism pathway genes were related to CDH4 in OSCC **F**. Correlation analysis showed that peroxisome pathway genes were related to CDH4 in OSCC **G**. Correlation analysis showed that ferroptosis related genes were related to CDH4 in OSCC **H**-**J** (* indicates *P* < 0.05, **indicates *P* < 0.01, ***indicates *P* < 0.001, ****indicates *P* < 0.0001, and Values are presented as means ± standard error.)

In OSCC, CDH4 is considered to promote the proliferation and metastasis of cells and play a role in the development of tumors. We used bioinformatics to analyze the database samples, hoping to find the related genes and pathways to explain the mechanism of CDH4. In order to explore suitable pathways, we chose to start with HNSCC for a larger sample size, and after verifying the corresponding pathways, we analyzed the correlation between the related genes of these pathways and CDH4 in OSCC. Moreover, we also analyzed the correlation between CDH4 and key genes of ferroptosis to find out the possible mechanism of CDH4. We found the top ten related pathways that correlated with CDH4 by GSEA in the Hallmark gene set in HNSCC (Fig. 6A). we selected three pathways that are most likely to be related to the previous experiments, such as the EMT pathway (Fig. 6B), fatty acid metabolism pathway (Fig. 6C) and peroxisome pathway (Fig. 6D). The EMT pathway was significantly positively correlated with CDH4 in OSCC. It had 13 genes positively correlated with CDH4 in EMT (Fig. 6E). The fatty acid metabolism pathway and peroxisome pathway were found many gens negatively correlated with CDH4 (Fig. 6F-G). CDH4 gene is also related to many key genes of ferroptosis in OSCC (Fig. 6H-J). These results indicated that CDH4 protects cell proliferation by resisting ferroptosis, while the promotion of cell mobility by CDH4 may be related to EMT pathway.

## Discussion

CDH4 encodes R-cadherin, which is a member of the cadherin family. The function of R-cad in tumors is controversial, and its role in OSCC is unknown. Some studies have shown that CDH4 is a tumor suppressor in nasopharyngeal carcinoma, gastrointestinal tumors, mammary tumors and lung cancer [18–22]. In another previous study, CDH4 was shown to be a gene that promotes cancer [23–27]. R-cadherin expression has been found in rhabdomyosarcomas and is absent in normal myoblasts [24]. In renal cell carcinoma, the expression of CDH4 is higher than that in normal kidney tissue [25]. By analyzing the TCGA database, the results show that CDH4 is more highly expressed in cancer tissue than in normal tissue in HNSCC and OSCC. Clinical OSCC samples also confirmed that CDH4 is highly expressed in cancer tissue. R-cadherin improves the motility of cells via GTPase activity in cervical cancer [26]. Our study also confirmed that CDH4 promoted the cells motility and proliferation in OSCC.

In osteosarcoma, CDH4 was shown to activate c-Jun via the JNK pathway and improve self-renewal ability to promote tumorigenesis and metastasis [27]. Tumor cells self-renewal ability play an important role in tumor survival, proliferation, metastasis and recurrence. Postoperative recurrence is an obstacle in the treatment of OSCC [54, 55]. we had the same finding about CDH4 in OSCC. CDH4 enhanced the self-renewal ability of tumor cells in OSCC.

Through GSEA of the Hallmark gene set, CDH4 was found positively correlated with multiple tumor-related pathways. In particular, it has a high correlation with the EMT pathway. EMT is a key pathway in tumor metastasis and recurrence. The sample size of OSCC is over 300. For the large sample data over 300, the samples with R value of correlation analysis above 0.1 and P < 0.05 are considered to have significant correlation. EMT is a classical and important pathway for cadherin to promote cell invasion and metastasis, and it is studied daily in tumors. Correlation analysis showed that 13 genes in EMT pathway were positively correlated with CDH4. EMT occurs because the expression of E-cadherin (CDH1 coding), which expresses epithelial characteristics, is suppressed, and the expression of N-cadherin (CDH2/CDH12 coding), which expresses mesenchymal characteristics, is increased [56]. Cells change from epithelial characteristics to mesenchymal characteristics, making it easier for cells to move [57]. The occurrence of EMT will cause the invasion and metastasis of tumor cells. EMT correlation analysis showed that CDH4 was positively correlated with FOXC2 and CDH2 gene. FOXC2 can inhibit the expression of E-cadherin, and N-cadherin has been proved to promote tumor in OSCC [58, 59].

After analyzing the genes related to CDH4, we found that except the above two genes, the other genes PLDO3, FSTL3, COLGALT1, FN1, PMEPA1, LGALS1, COL4A2, SERPINH1 and SPOCK1 were all found to promote the occurrence and development of different tumors [60–68]. Because there is a positive correlation between CDH4 and these genes, we think that CDH4 may plays a positive role in EMT, which affects the movement and invasion of tumor cells through EMT pathway.

Some studies point out that there is a relationship between cadherins and ferroptosis. E-cadherin activates the Merlin and Hippo signaling pathways to suppress ferroptosis. Cancer cells with epithelial properties are less sensitive to ferroptosis than those with mesenchymal properties [69]. VE-cadherin can resist the destruction of blood–brain barrier cells caused by iron metabolism disorder [70].

According to the results of previous experiments, we think that the change of cell proliferation ability caused by CDH4 is because it resisted the sensitivity of cells to ferroptosis. Ferroptosis is caused by iron metabolism abnormality and two redox systems (lipid peroxidation and thiols) abnormality are the causes of iron-dependent ROS accumulation in cells [33]. Experiments confirmed that the raising consumption of GSH and GPX4 and the increase of intracellular lipid peroxidation occurred after the decreasing of CDH4 in OSCC. We used enrichment analysis show two correlation pathways: fatty acid metabolism pathway peroxisome pathway. We verified that the related genes of these two pathways of CDH4 were negatively correlated in OSCC. We analyzed the correlation between CDH4 and ferroptosis marker genes, ferroptosis suppressor genes and ferroptosis driver genes in OSCC. Data shows that half of marker genes (GPX4, FTH1, TFRC, PTGS2) of ferroptosis were correlated with CDH4, and the CDH4 was positively correlated with the genes (FZD7, FIDC5, RARRES2, ATF2, HSPA5, USP11, KLHDC3, PLIN2, ACOT1, MLST8, RBMS1) that inhibit ferroptosis and negatively correlated with the genes (AQP3, NOX5, DUOX1, DUOX2) that promote ferroptosis.

## Conclusions

In summary, CDH4 effectively promotes the proliferation, invasion and metastasis of OSCC cells. It may enhance EMT pathway and reduce the sensitivity of OSCC cells to ferroptosis. CDH4 is a therapeutic target in OSCC.

## Supplementary Information

**Additional file 1.**

## Data Availability

All data generated or analyzed during this study are included in this published article.
