# Supplementary material for: CDH4 inhibits ferroptosis in oral squamous cell carcinoma cells
Source: BMC Oral Health. 2023 May 27;23:329. doi: 10.1186/s12903-023-03046-3 (PMC10224206; doi:10.1186/s12903-023-03046-3)
Supplement: Supplementary file 1 — Additional file 1. [file 12903_2023_3046_MOESM1_ESM.pdf]

Figure 2.

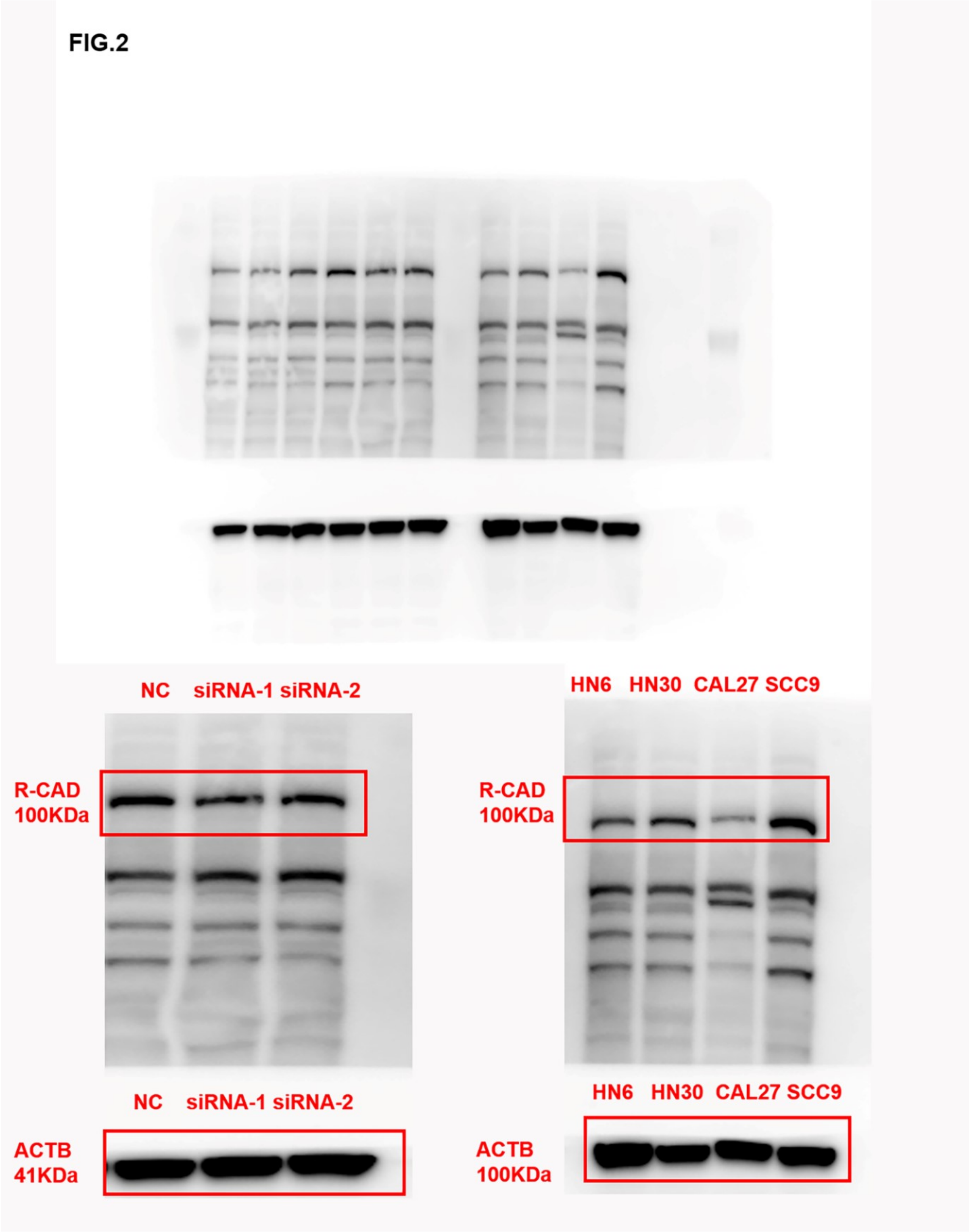

Figure S1. The original Western Blot images for R-Cadeherin and ACTB in different cell lines (right, related to Fig 2B) and in HN30 cells (left, related to Fig 2B).

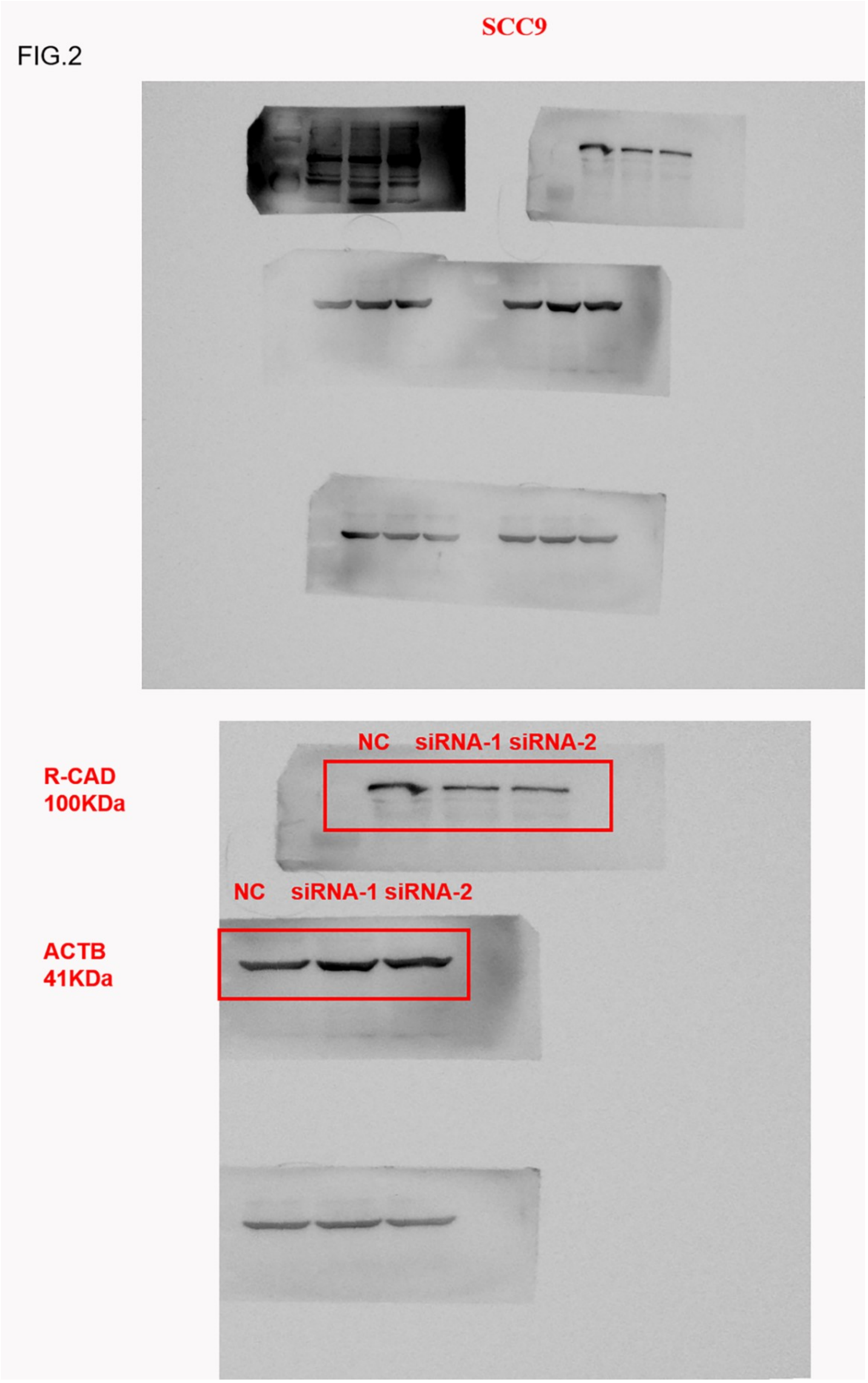

Figure S2. The original Western Blot images for R-Cadeherin and ACTB in SCC9 cells (related to Fig 2F).

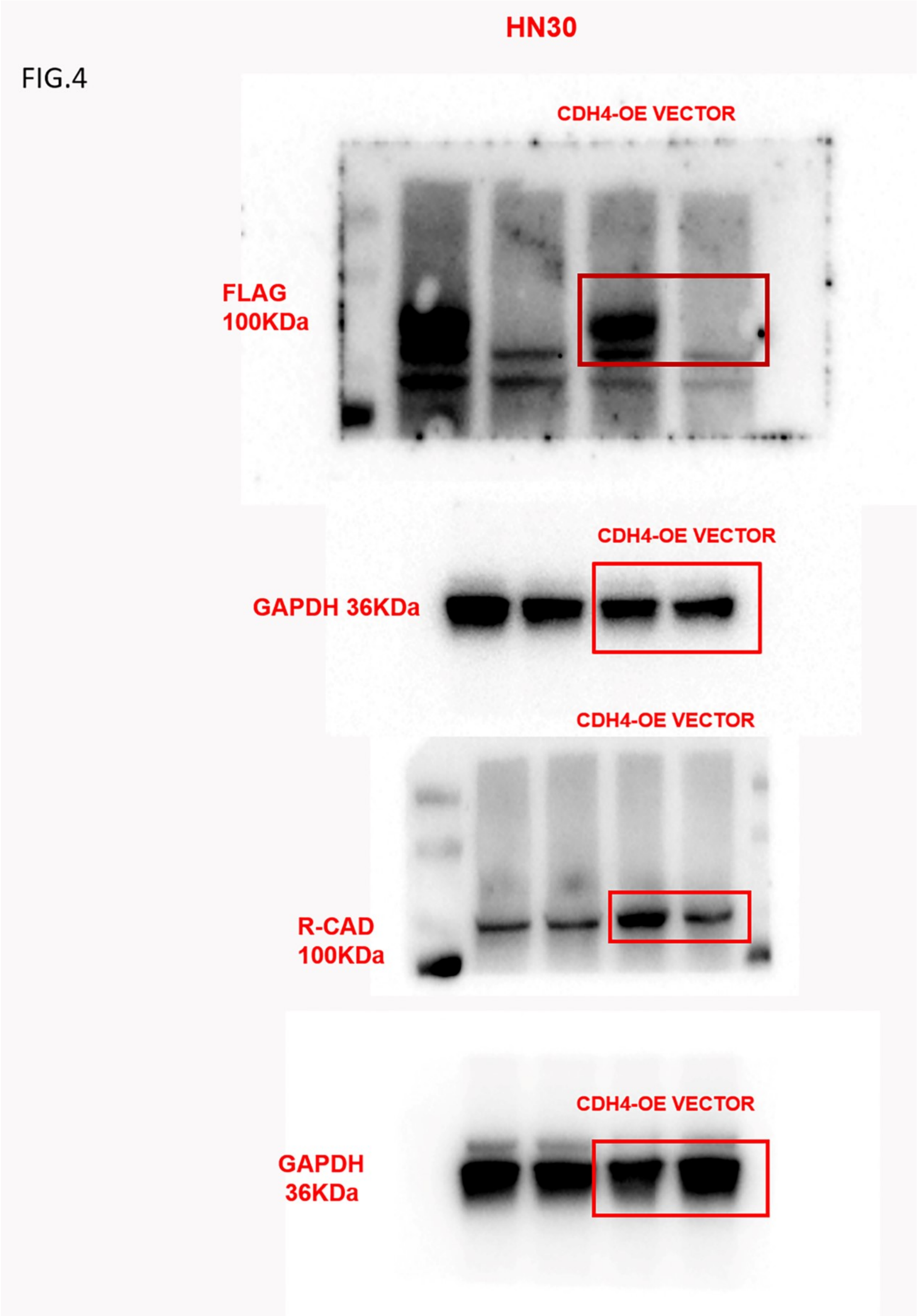

Figure S3. The original Western Blot images for R-Cadeherin and GAPDH in HN30 cells (related to Fig 4A).

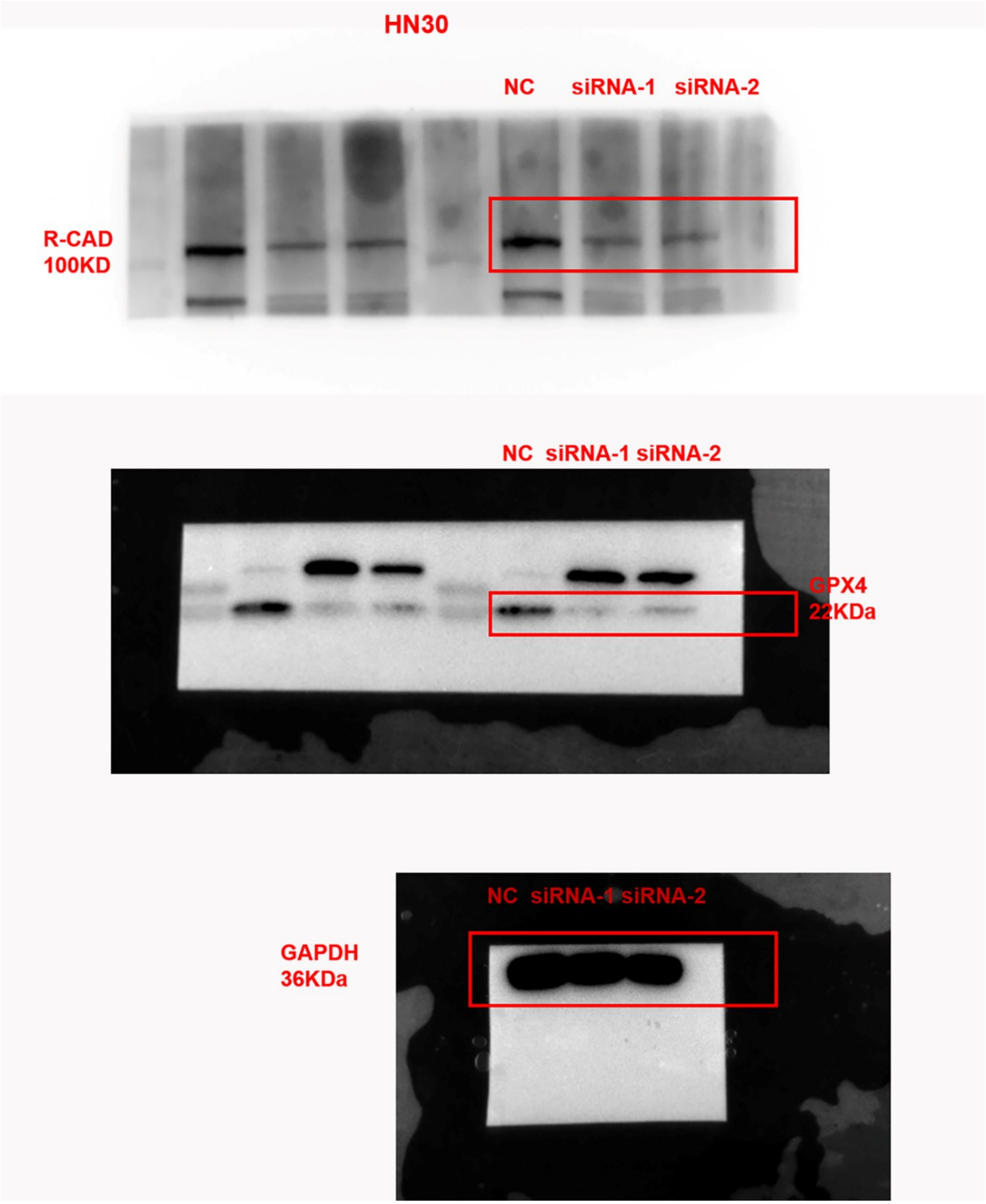

Figure S4. The original Western Blot images for R-Cadeherin, GPX4 and GAPDH in HN30 cells (related to Fig 5E).

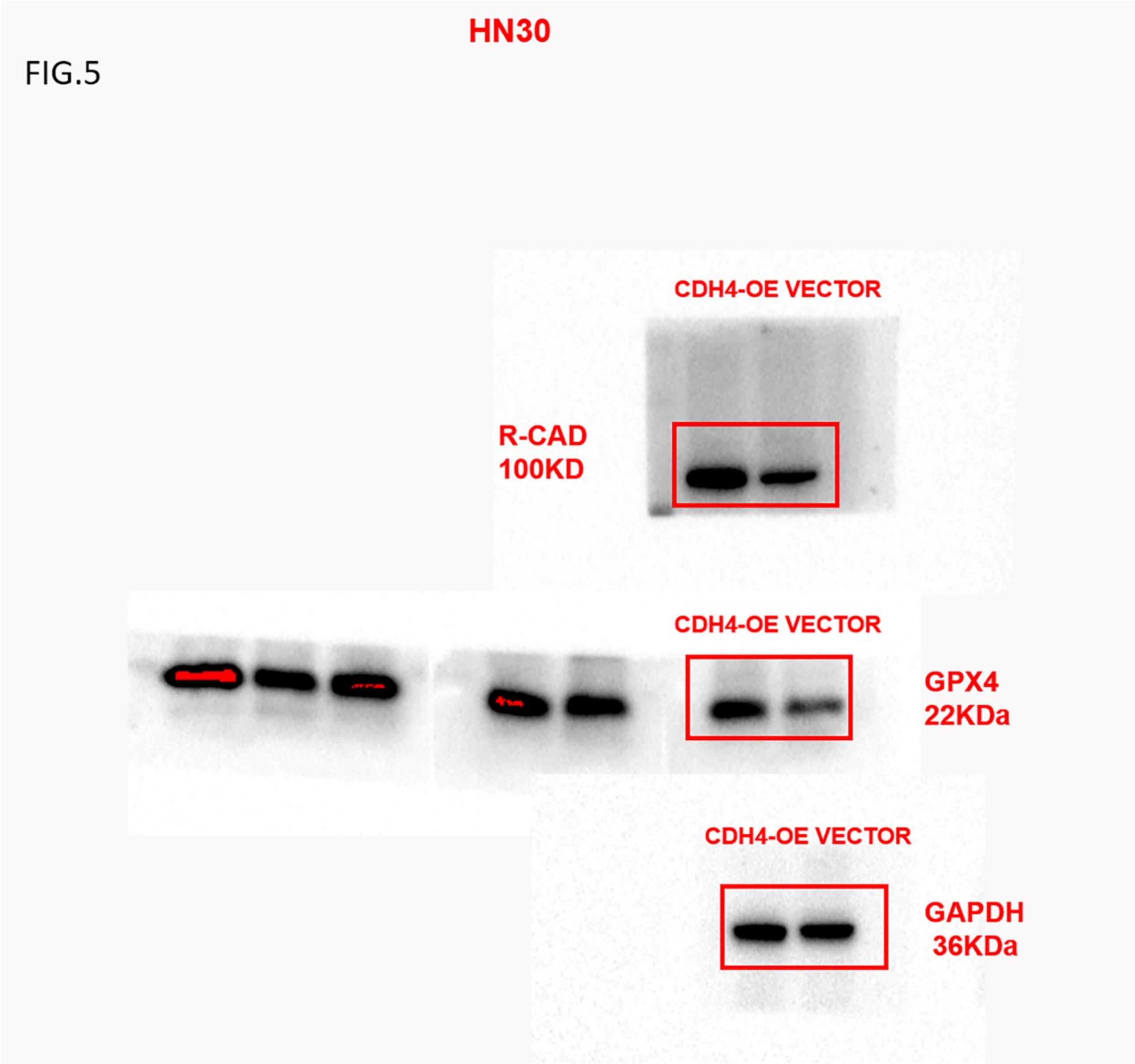

Figure S5. The original Western Blot images for R-Cadeherin, GPX4 and GAPDH in HN30 cells (related to Fig 5F).

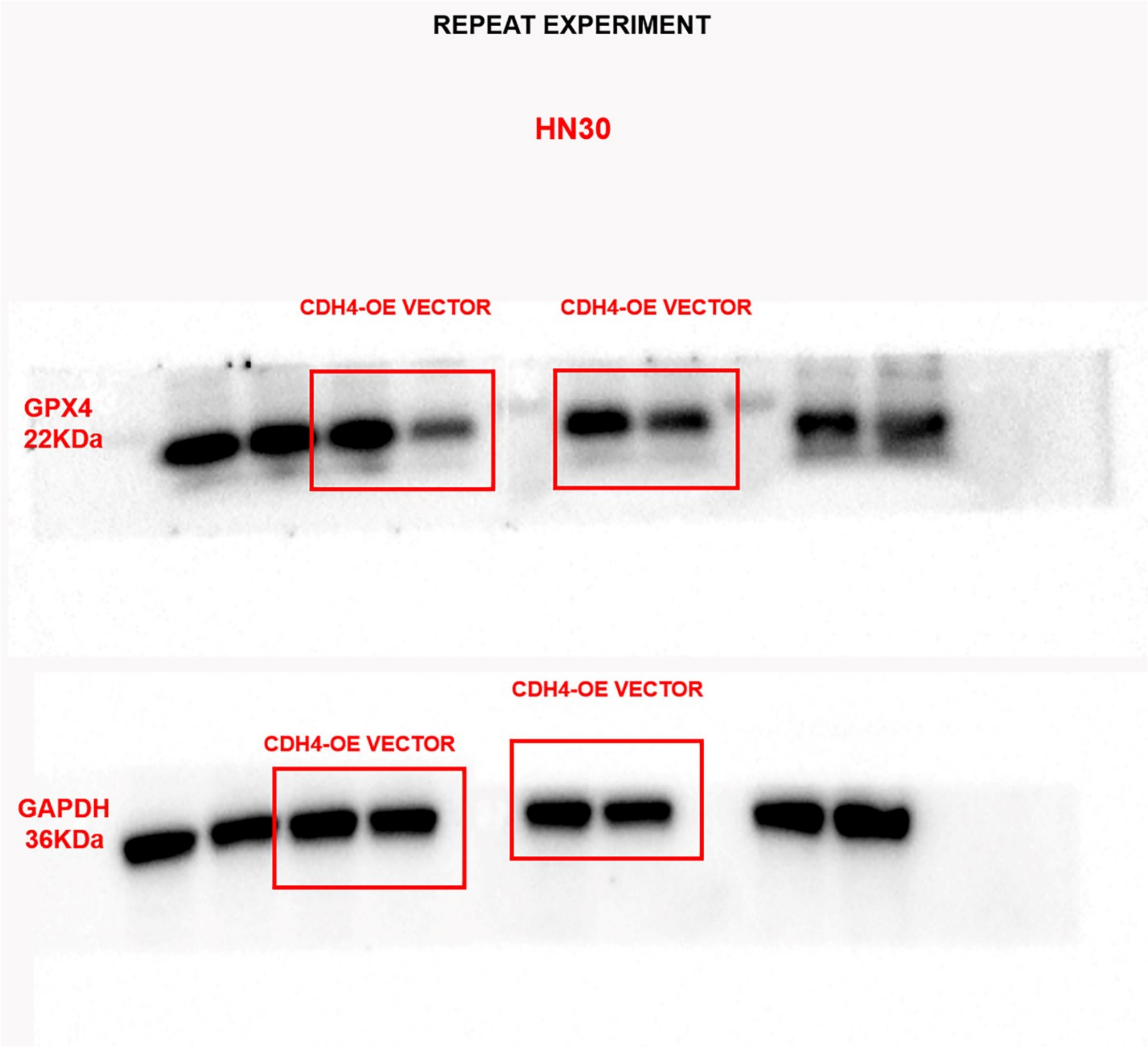

Figure S6. The original Western Blot images of repeat experiments for GPX4 and GAPDH in HN30 cells (related to Fig 5F).

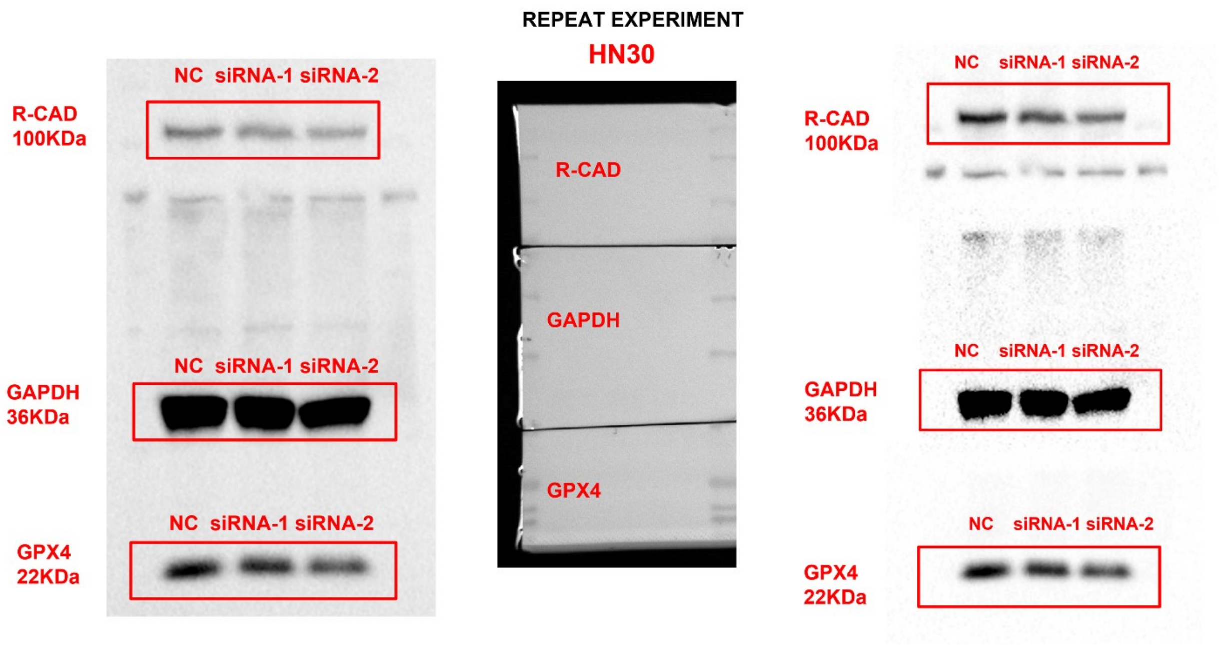

Figure S7. The original Western Blot images of repeat experiments for R-Cad, GPX4 and GAPDH in HN30 cells (related to Fig 5E).

Figure 5, repeated.

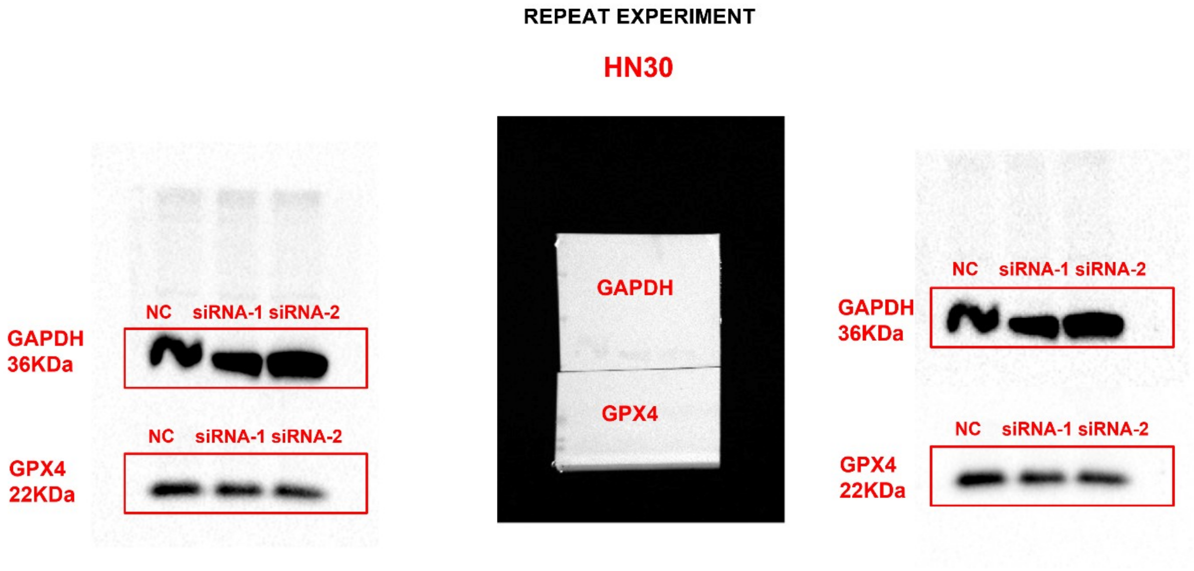

Figure S8. The original Western Blot images of repeat experiments for GPX4 and GAPDH in HN30 cells (related to Fig 5E).

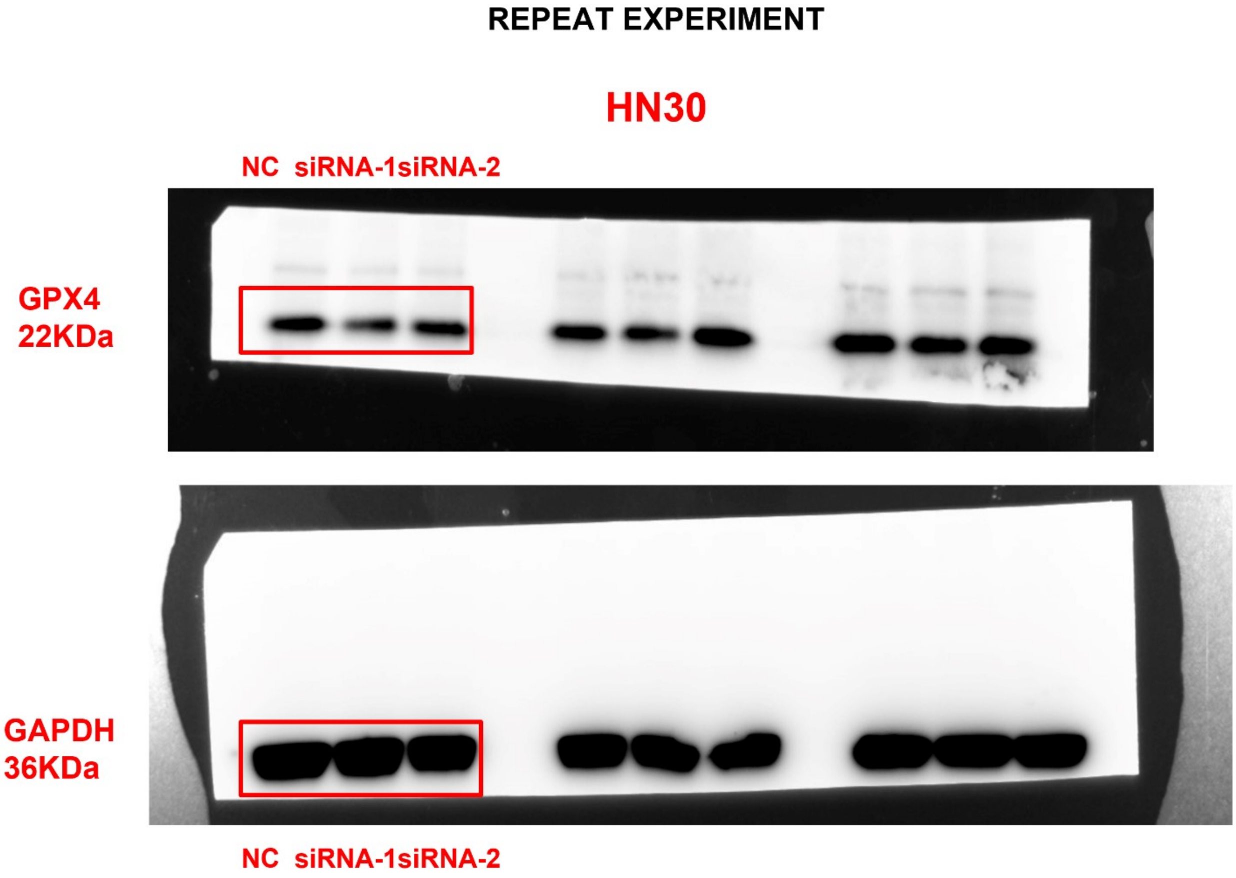

Figure S9. The original Western Blot images of repeat experiments for GPX4 and GAPDH in HN30 cells (related to Fig 5E).
